# Global Survey of Microscopy Core Facilities

**DOI:** 10.1111/jmi.70097

**Published:** 2026-05-07

**Authors:** Kirk J. Czymmek, Alexander Scott, Jessica Back, Derek Davies, Zhenxi Gao, Masilamani Elangovan, Peter O’Toole

**Affiliations:** 1 Advanced Bioimaging Laboratory, Donald Danforth Plant Science Center, 975 North Warson Road, Saint Louis, Missouri 63132 USA; 2 Department of Biology, University of York, Wentworth Way, YO10 5DD, United Kingdom; 3 Microscopy, Imaging, and Cytometry Resources Core, Karmanos Cancer Institute, Wayne State University, 615 HWCRC, 4100 John R, Detroit, MI 48201 USA; 4 Idea Elan, LLC, 13800 Coppermine Rd, Herndon, VA 20171 USA; 5 School of Life Sciences, Peking University, No. 5, Yiheyuan Road, Haidian District, Beijing, 100871 China; 6 The Francis Crick Institute, 1 Midland Road, London NW1 1AT United Kingdom; 7 Imaging and Cytometry Bioscience Technology Facility, Department of Biology (Area 15), University of York, York, YO10 5DD United Kingdom

**Keywords:** Microscopy Survey, Core Microscopy Facility, Longitudinal Study, User Fees, Facility Management

## Abstract

As part of two open international core facility surveys focused on microscopy and flow cytometry, we obtained an in-depth snapshot of their state-of-play. Here we focus on microscopy cores across “The Americas”, “Asia Pacific” and “Europe & Africa” with responses from 241 facilities. We compared instrument types and numbers, staffing, user fees, funding, etc. This revealed both remarkable similarities and notable differences. For example, the staff/instrument ratio in Europe & Africa was smaller compared to other regions World View and Asia Pacific had a larger proportion of electron microscopes. Importantly, we want to emphasize that the anonymized raw data is made available for anyone to do their own further mining and analysis, including cross-correlations and constrained groupings. This survey will serve as an essential baseline for future longitudinal studies following trends across all metrics as our facilities evolve.

## Introduction

Many core facilities (sometimes referred to as shared resource laboratories) generally operate with limited knowledge of how they compare with others of similar size, similar institutes, and/or geographical region. Over the years, many of these facilities have benefited from *ad hoc* or structured surveys specific to their core competencies. For example, the Association of Biomolecular Resource Facilities (ABRF) has several valuable broader surveys including job satisfaction, compensation, and benchmarking^1–4^ or a Federal Ministry of Education and Research (BRBF, Germany) funded study of research quality in core facilities ^5^, as well as many other independent surveys^6–9^. By developing the framework for a longitudinal study we hope to standardize the questions and make comparisons over time possible. We also hope that other groups running future surveys will follow suit noting some of our discussions around potential future improvements. Ultimately, our colleagues serving as coordinators, managers and directors must periodically report progress and justify value/need to administrative and scientific oversight committees. Furthermore, cores are not, or should not, be static and ideally evolve to support their institutions’ research programs, scientific need and as technological advancements occur. With this in mind, we developed a matched survey for both microscopy and flow cytometry^10^ communities to obtain a snapshot of core facilities of these closely related and most commonly found single or combined core facilities. We surveyed common operational and usage metrics, including supporting personnel resources (staff and instruments) and will repeat this survey periodically over time with predominantly the same questions and adjusted for trends and the needs of the community and allow longitudinal changes to be identified. This survey will allow core facilities to benchmark against their global or regional peers and provide important information in these comparisons to support conversations with national funders, local institutional management/oversight committees and commercial manufacturers. We asked a comprehensive set of questions to make the data as useful to as many groups as possible, and to help future proof subsequent surveys to follow trends that may become important.

The microscopy survey captured data between September 2020 and February 2021, publicized via various networks, including the Confocal and Microscopy Listserv’s, Bioimaging UK, European Light Microscopy Initiative, Light Microscopy Australia, Bioimaging North America, and various social media accounts that helped cascade awareness, attracting responses from 241 different core facilities. Although the geographic spread was wide, some countries were represented by only one facility. To determine if there were any geographical differences in the responses, we have grouped the data into three global regions: The Americas (North and South America), Europe and Africa, and Asia Pacific (Asia and Australia). Unfortunately representation from lower income regions was naturally fewer making it hard to draw any local conclusions, but these have been grouped to ensure that everyone’s data has contributed. We note that the anonymized raw data has been made available for deeper analysis. Further granularity for reporting here would lead to too few data points to be significant and risk specific facilities being individually identifiable.

The anonymized raw data is available in spreadsheet form to allow further correlations that were not addressed in this report, which has focused on broad global trends (Supplementary Table 1). This is the first dataset of the planned series to allow for temporal evaluation of changes that take place in microscopy and flow cytometry core facilities operations and instrumentation over time. From a historical perspective, this survey also included questions about the response and impact of COVID-19.

## Results

### Demographics

**Figure 1:**
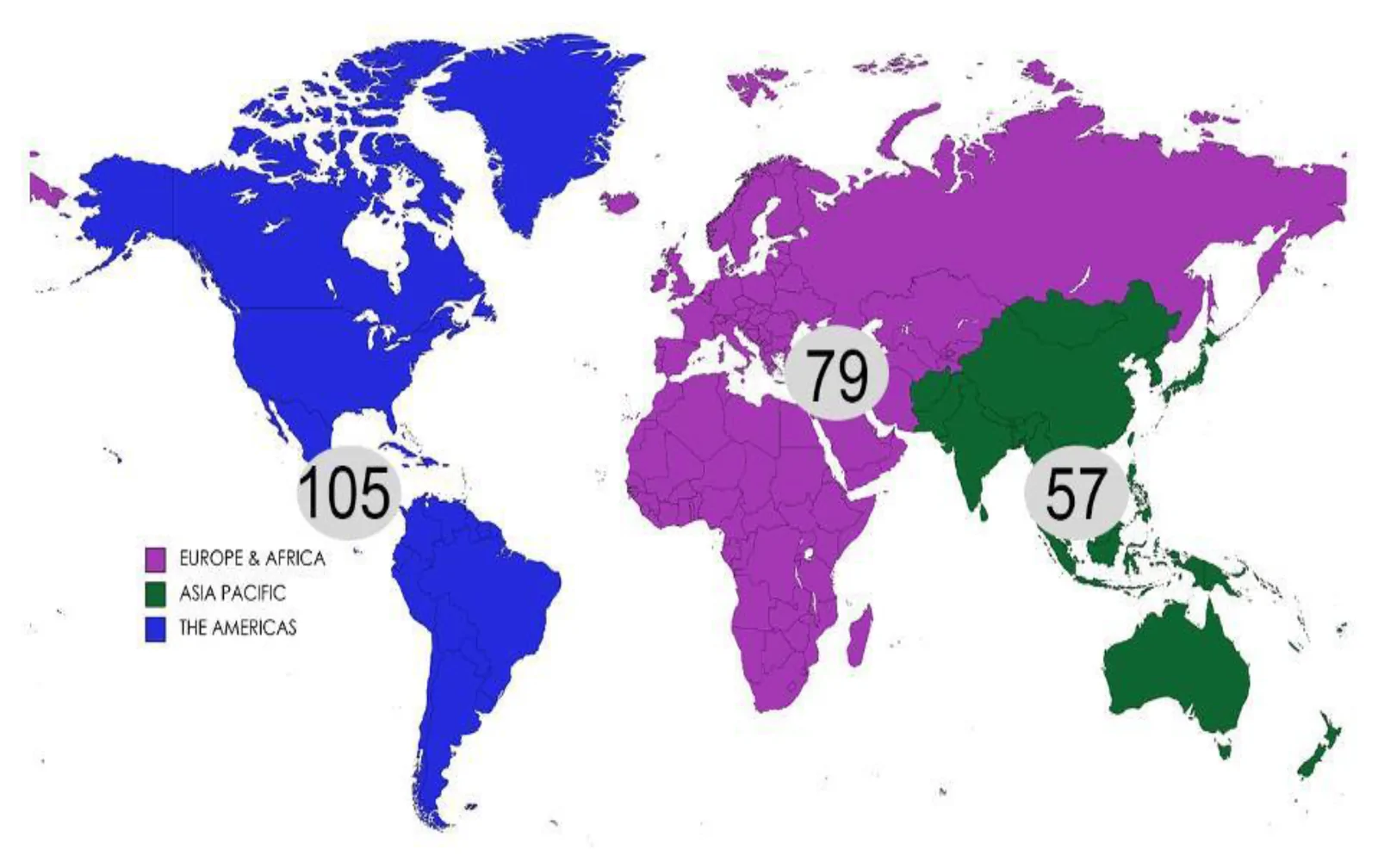
Microscopy Survey Demographics. This global survey included 241 participants from Microscopy core facilities from three geographical regions: The Americas at 43.57% (blue), Europe and Africa* at 32.78% (magenta), and the Asia Pacific at 23.65% (green). *Note, Europe and Africa region (magenta) includes Russia (0 participants) and the Middle East (4 participants). See Supplementary Figure 1 to see global core microscopy facility distribution heatmap (by country).

**Figure 2:**
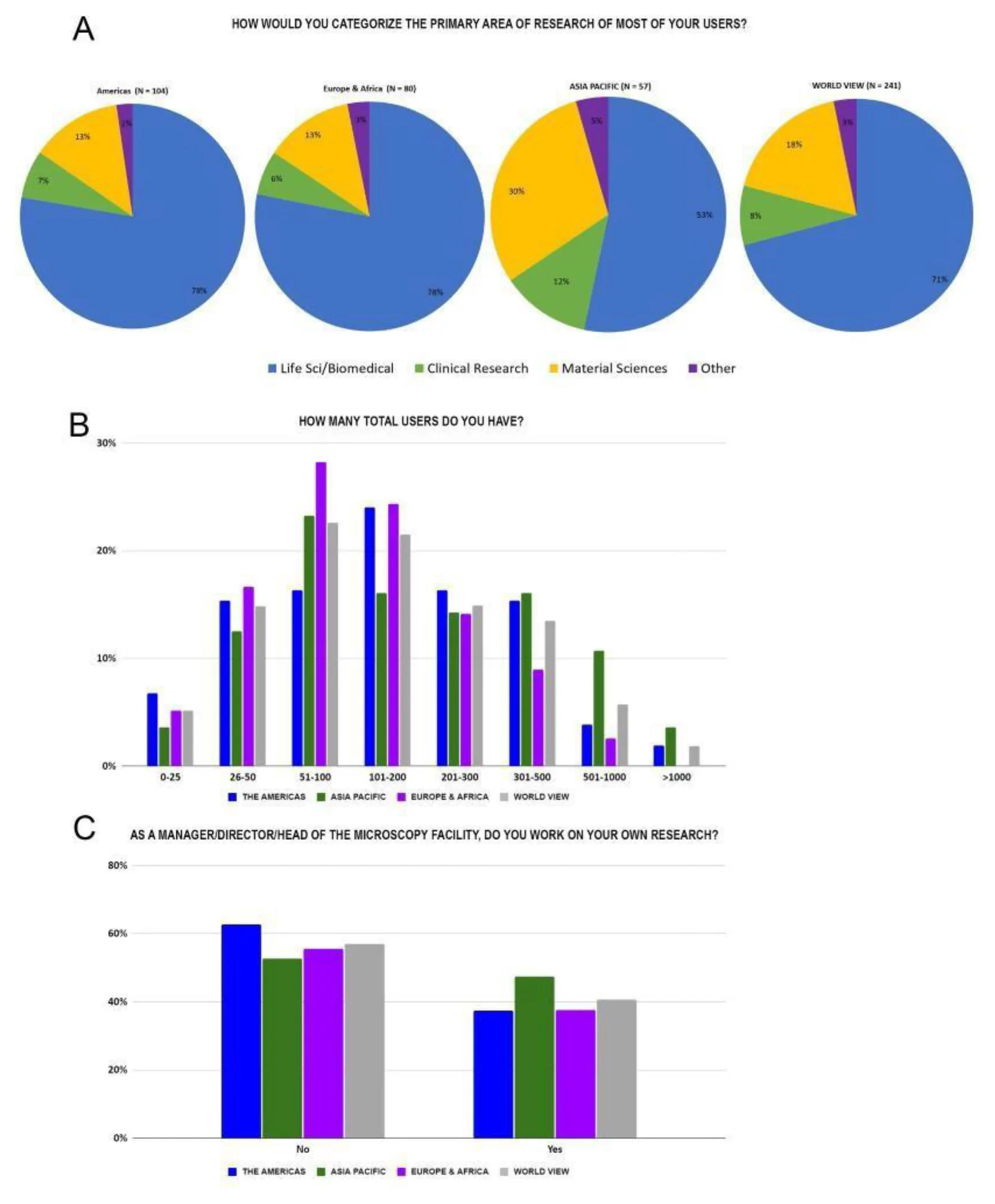
Scientific Emphasis, User size and remit across regions (A) Pie Charts of the primary area of research for users, (B) Bar graph of distribution of total users (C) Bar graph of Manager/Director ability to do own research.

This survey captured responses from 241 participants from Microscopy core facilities with good representation of the three geographical regions: The Americas (105, 43.57%), Europe and Africa (79, 32.78%), and the Asia Pacific (57, 23.65%) (Fig. 1). The “World View” is the combined data from all regions and we also include a global country-based distribution heat map to allow readers to inspect the composition of each region (Supplementary Fig. 1). There was also a good spread of user facility focus, and while Life Science/Biomedical was the dominant primary area of interest (Question 2, “How would you categorize the primary area of research of most of your users?”) (71 % World View, Fig. 2A), this was largely because the group was the easiest to reach via larger microscopy and cytometry societies. However, it was noted that many core facilities supported research across scientific disciplines with 18 % having Material Science users, which was notable greater in the Asia Pacific (30%). When it came to the number of users (Question 8, “How many total users do you have?”) supported annually in core facilities, the World View maximum frequency was 51-100 users with 23% of cores including both Europe & Africa (28%) and Asia Pacific (23%), while the maximum frequency for The Americas was 101-200 users at 24% (Fig. 2B). To understand the ability of core managers to work on their own research rather than strictly service responsibilities (Question 9, “As a manager/director/head of the Microscopy Facility, do you work on your own research?”), 57% of lab heads responded affirmatively in the World View, with The Americas (63%), followed by Europe & Africa (56%) and Asia Pacific (53%) which was nearly evenly split (Fig. 2C). We asked the question “What is the annual operating cost of your Microscopy Facility?” (Supplementary Fig. 2, Question 10) and noted a bimodal distribution with the World View peaking at $101K-$200K/annum and >$1M, both with just a little more than 20% or respondents. However, only 62% included staff salary in their calculation (Supplementary Fig. 2, Question 11, “Does the total operating cost from the above question include staff salary?”) and thus, underestimates the aggregate true cost of many core facilities and may also indicate that some institutions subsidize all or part of their Microscopy Facility staff salaries.

### Number and Types of Instruments

**Figure 3:**
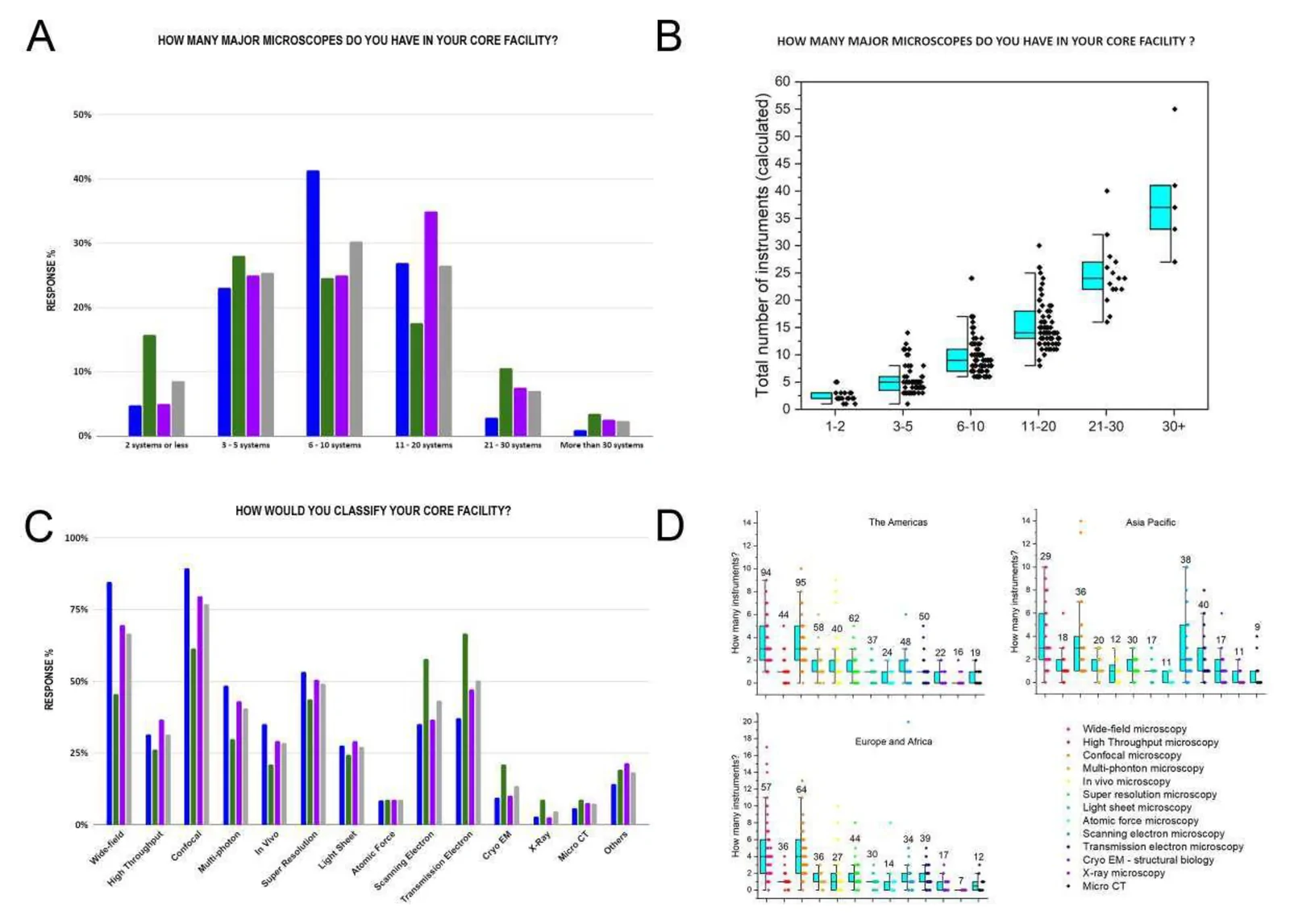
Major Microscope Metrics. (A) Bar graph of major microscope number. (B) Scatter plot of how many major microscopes. (C) Bar graph of microscope types. (D) Regional scatter plot showed distribution of major microscopes.

As expected, most core facilities had multiple microscopes, with a large majority having between 3 to 20 instruments and the highest frequency being Europe & Africa at 11-20 systems, World View and The Americas at 6-10 systems, and Asia Pacific at 3-5 systems/core (Fig. 3A, Question 4, “How many major microscopes do you have in your Core Facility?”). Wide-field and confocal microscopy were the most common instrument types in each core facility, followed by Super-Resolution Microscopy (SRM), and Electron Microscopy (EM), with other instrument types being much less common. Interestingly, a closer inspection of Question 5, “How many of each type of instrument?” (Supplementary Table 1), we determined that over 50% of all respondents (122/241) had at least one SEM, TEM or Cryo EM and 45% or all facilities having at least one LM also had at least one SEM, TEM or Cryo EM. This somewhat surprising high level of mixed platform composition of so many facilities, may in part reflect increasing consolidation across many institutions.

**Figure 4:**
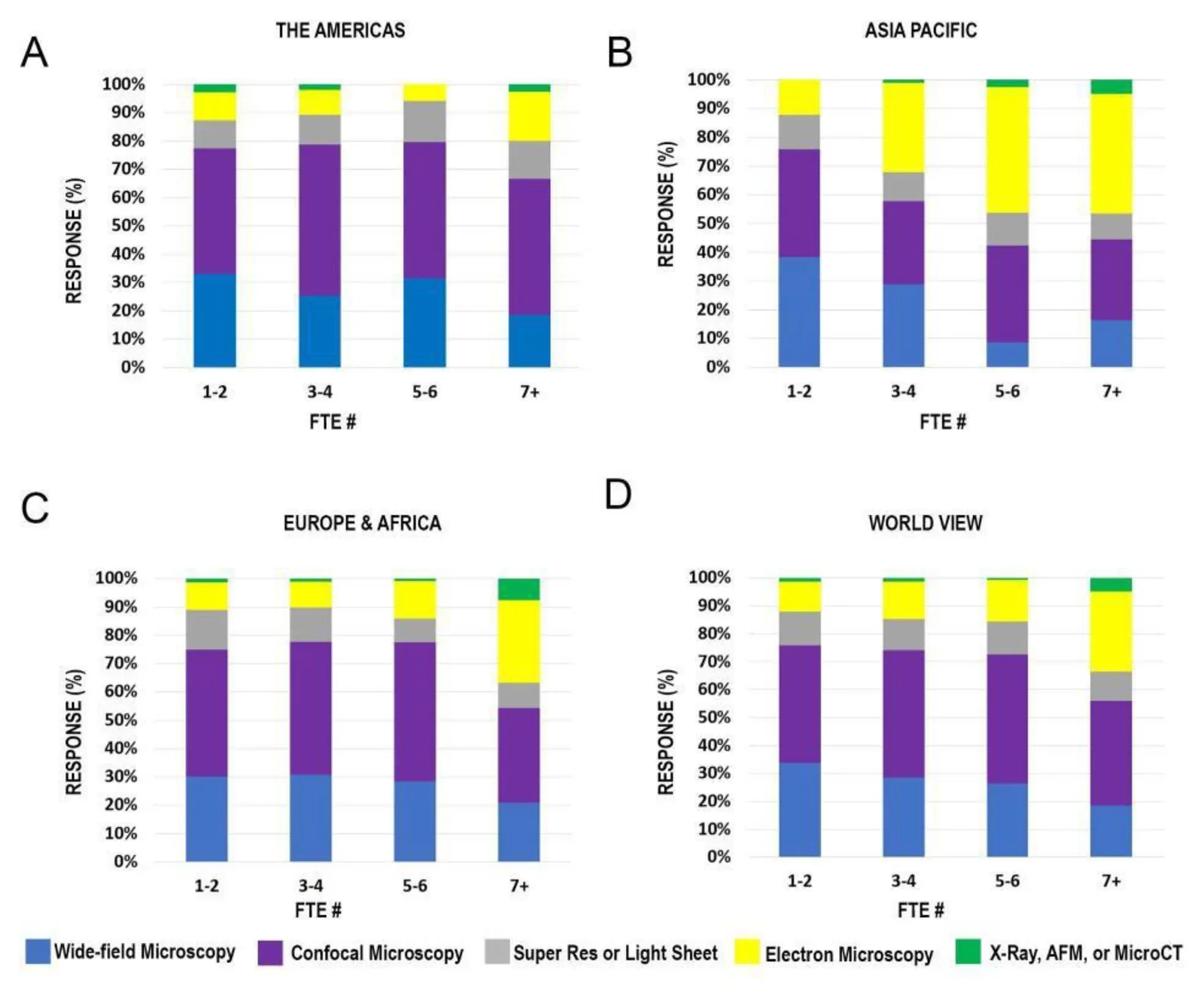
Instrument Type versus Full Time Equivalent (FTE). (A) Bar chart of The Americas. (B) Bar chart of Asia Pacific. (C) Bar chart of Europe & Africa. (D) Bar Chart of World View.

The World View trend was very similar with regards to the ratio of instruments to staff (Fig. 4), with the exception of the Asia Pacific which proportionally had more electron microscopes at increased staffing levels (3+ staff). It was notable that the ratio of wide-field, confocal, and SRMs in The Americas and Europe & Africa for up to 3-4 staff was similar and as the number of staff increased to 5 and greater, these labs trended to be more diverse in their offerings, with an increase in EM, and decrease in wide-field microscopy (Fig. 4). From a World View perspective, there were approximately 2 confocal microscopes and SRMs combined to every wide-field microscope, suggesting that core facilities mostly focus on high-end instrumentation, while lower cost instrumentation are probably owned by individual research labs rather than cores (Fig. 3C). It will be very interesting to see how the more niche or newer technologies trend over the coming surveys and whether platforms like cryo-EM, volume-EM, SRM and light sheet microscopy start to become more common across facilities or remain restricted to specialist and regional core facilities.

### Staffing

#### Number of Staff versus Instruments and Users

**Figure 5:**
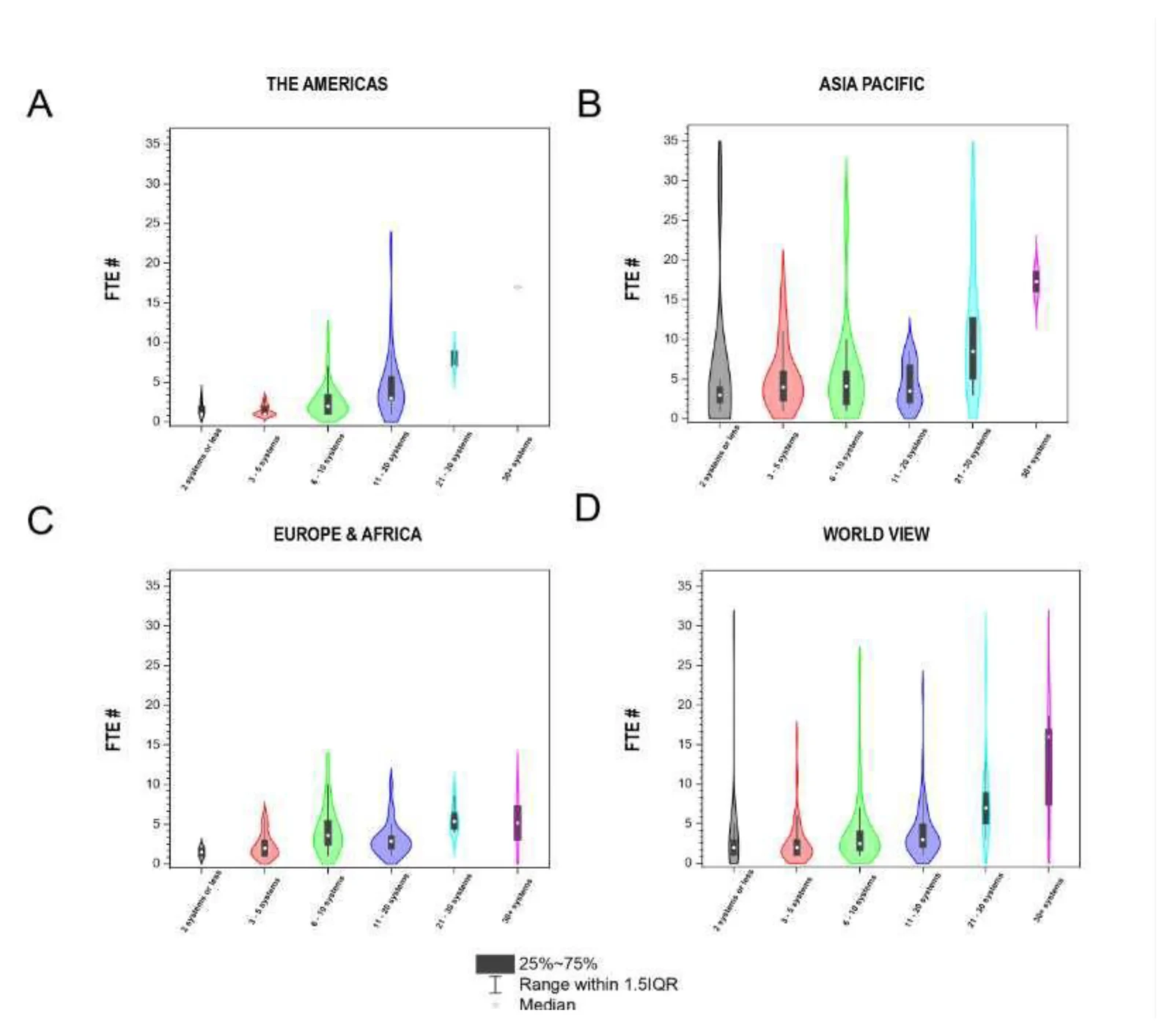
Full-Time Equivalent (FTE) Versus System Number Metrics across the Regions. (A) The Americas (B) Asia Pacific. (C) Europe & Africa. (D) World View.

**Figure 6:**
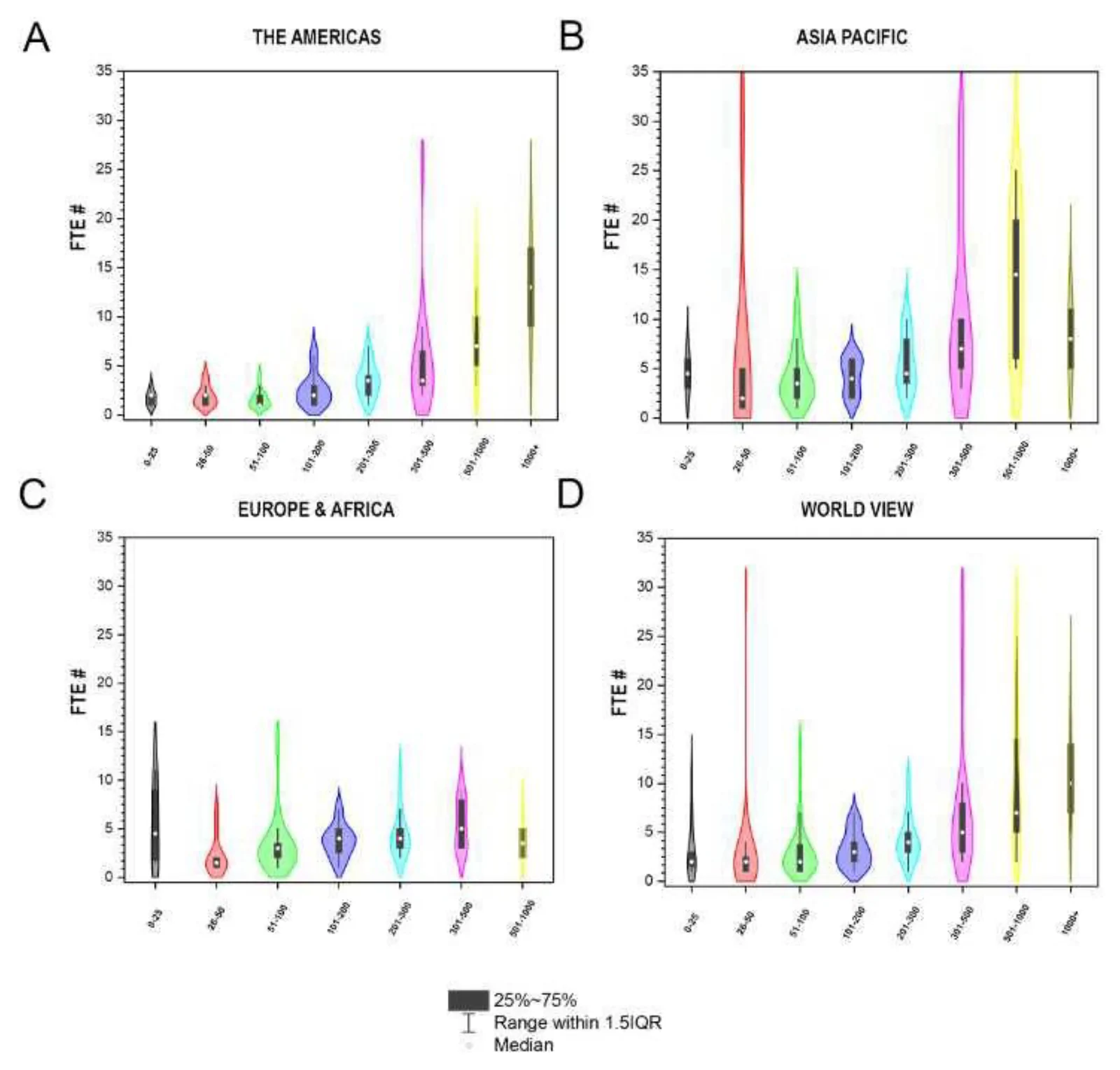
Full-Time Equivalent (FTE) Versus User Number Metrics across the Regions. (A) The Americas (B) Asia Pacific. (C) Europe & Africa. (D) World View.

**Figure 7:**
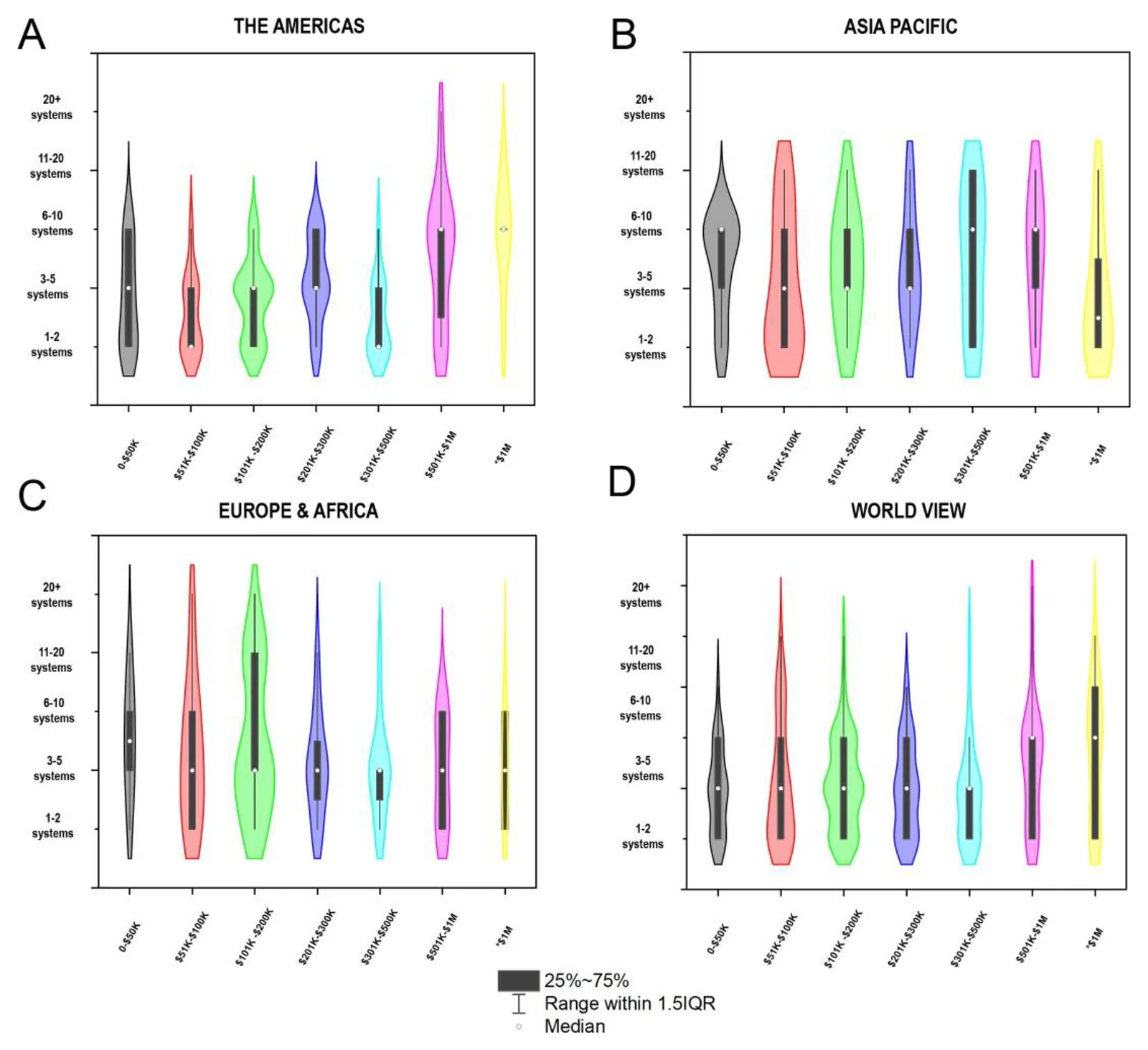
System Number Versus Revenue Metrics across the Regions. (A) The Americas (B) Asia Pacific. (C) Europe & Africa. (D) World View.

As expected, the staffing numbers increased relative to the number of instruments within the core facility (Fig. 5, Question 6, “How many Staff members do you have (excluding you)?” versus Question 5, “How many of each type of instrument?”). For this analysis we added one staff member to include the facility manager. Unfortunately, the specific number of how many staff members are needed per number of instruments was difficult to extract as the range was very broad and more likely reflected the instrument complexity and the funding models that support individual core facilities, rather than the total number of instruments within the facility. As a general trend, as calculated from the slope, The Americas had 3.4 instruments/staff member, Asia Pacific 1.8 instruments/staff member, while Europe & Africa respondents had the most instruments/staff member at 4.1, with a global average of 3.1 instruments/staff member (Supplementary Fig. 3). These ratios are remarkably similar to that found in the Schmidt^11^. It must be noted, however, that there were a few labs that had a far greater number of total instruments compared to staff members (Fig. 5) and vice versa (e.g. a lab with 2 instruments was being supported by 30 staff) that impacted the metrics. Also, since the survey did not accurately capture the number of Full Time Equivalents (FTE) (likely as we did not define this term for regions less familiar with its use) the total number of staff could be a result of more part-time workers in the labs being reported as FTE and thus more staff per instrument. It could also be that more Europe & Africa labs also host cytometers or other non-microscopy instruments that were not captured within this survey, and thus additional staff would be a result of other non-measured equipment and/or services. Additionally, it could also reflect that the core labs in different regions offer full-service support versus models that emphasize users trained to work independently. The complexity of the instruments also dictated the number of support staff. For example, the ratio of SRM verses confocal microscopes tended to stay constant in the Americas, where the staff number was greater, while in Europe and Africa, the proportion of confocal microscopes was greater relative to the number of SRMs. Thus, the instrument/staff ratio does not necessarily increase in a predictable way simply by knowing the total number of instruments as reflected in the slope (Supplementary Fig. 3) and discussed above. Nevertheless, taken together, these instrument/staff member ratios still can serve as a very useful guide for all cores, and especially in the event that some core facilities deviate substantially from the World View of about 3 instruments/staff member, or ~2 instruments/staff member in an EM concentrated facility. Parsing the number of FTE versus the number of users (Fig. 6), there was little FTE change in facilities having 0-200 users (~2-3FTE) but the number of FTE increased with facilities having at least 300 users (~5FTE) to facilities with 1000+ users (~10 FTE) in the World View. Interestingly, the World View number of systems versus revenue was largely flat (3-5 system) up $500K USD and cores with $500K+ USD in revenue had an average of 6-10 systems (Fig. 7). Overall, this data likely reflects the sheer diversity in types of cores, microscope composition, institutional cost recovery expectations and their user base size, to name a few.

#### Annual Operating Costs versus Number of Instruments or Staff

**Figure 8:**
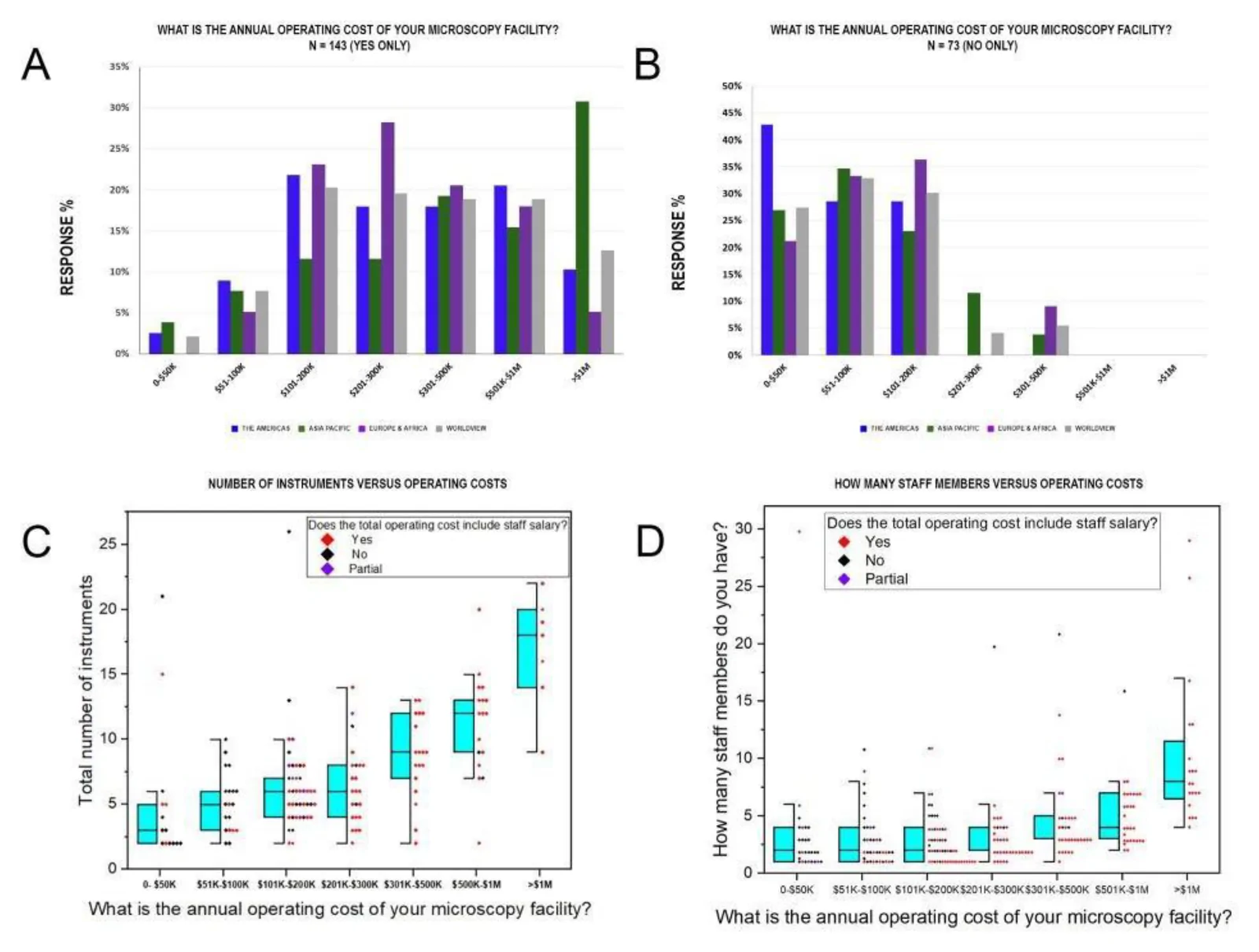
Annual Operating Costs Metrics (A) Operating costs across regions (B) Did operating costs including staff salary (C) Operating Costs with salary versus total number of instruments (D) Operating Costs with salary versus staff.

**Figure 9:**
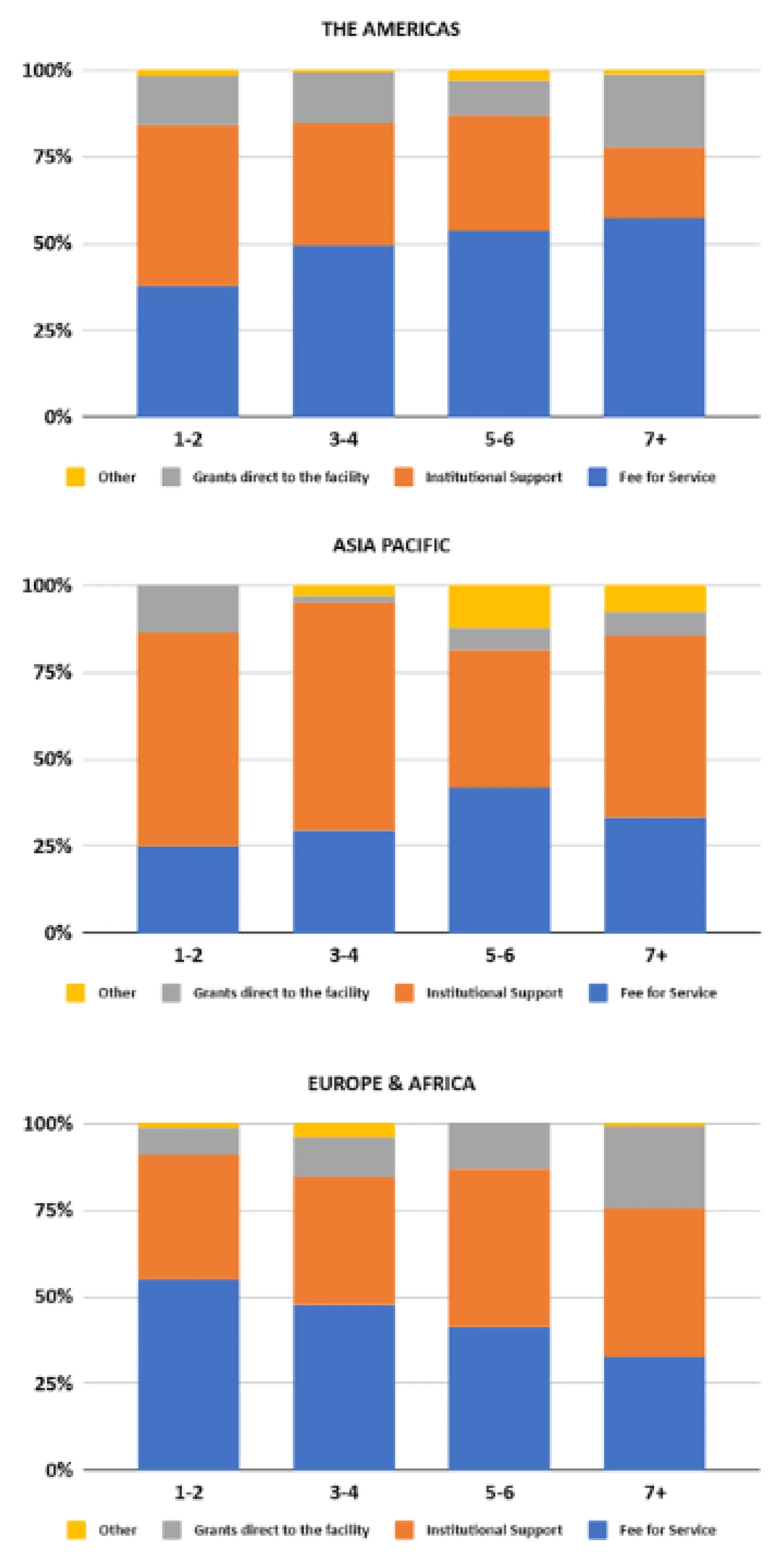
Funding Sources Metrics across the Regions versus Staff Number. (A) The Americas (B) Asia Pacific. (C) Europe & Africa.

When considering all respondents, t he global annual operating costs of each core (all currencies converted to US Dollars) were spread fairly evenly throughout the survey range (Supplementary Fig. 2, Question 10), with a peak at $101-200K USD and little difference between the regions with regards to the spread of operating costs per lab. It was notable that the Asia Pacific had the majority of lowest cost and highest cost facilities when looked at collectively (Fig. 8). However, in Question 11 (Supplementary Fig. 2), we asked if the operating costs reported in Question 10 included salary, since many cores do not account for this in their budget models. In general, we assumed that most labs with lower annual running costs did not include salaries, whereas the higher end almost exclusively included staff costs. Thus, to get a better sense of true operating costs for those who reported all costs, we separated and plotted the surveys that did include salaries (Fig. 8A) versus those that did not (Fig. 8B). For yes respondents, operating costs for The Americas and Europe & Africa had ~ 20% in each bin from $101K - 1M USD with their peaks being $101-200K USD and $201-300K USD, respectively. China, notably trended upwards across the operating cost range with a peak of over 30% of respondents with greater than $1 million USD, probably reflecting the higher proportion of EM. In The Americas, and Europe & Africa, income sources were split fairly evenly between fee-for-service (being slightly greater) and institutional support, with grants direct to core facilities being the least common source of income (Fig. 9). In contrast, the Asia Pacific showed a much higher proportion of direct institutional support and it will be interesting to see if these trends in funding sources will hold true in future surveys.

Overall, the funding sources were remarkably similar (World View 36.2% Fee for Service, Institutional Support 34.5%, 21.2% Grants, 8.1% Other) (Supplementary Fig. 2, Question 13, “What percentage do you receive from the following sources of funds?”), but a closer look showed these funding sources (Fig. 9) had notable regional differences. Specifically, when the number of staff within a core facility was considered, in The Americas and Asia Pacific, fee-for-service increased in proportion with increased staff numbers, whereas Europe & Africa showed the opposite trend with an increase in the proportion of direct grants to the core facilities with 7+ staff. For Europe & Africa and The Americas, this switch to institutional support in core facilities with higher staffing numbers could reflect larger national centers (e.g. EMBL, NIH, MRC) that are funded directly by charities or national/international funders, and our survey did not differentiate these facilities from those that are set-up within academic institutions or commercial labs.

#### Instrumentation

In 2019, a large majority of core facilities were seeing utilization increase with a significant proportion having over 25% (Supplementary Fig. 4 A. Question 43, “How did your utilization change in the last 3 years up until the end of 2019?”). This is encouraging and could reflect that the popularity, value, and/or size of core facilities were still growing pre-COVID. Probably one of the most important aspects of Microscopy Facility management is setting a fee structure which needs to balance the budget requirements of cost recovery, local/regional competition, funding agency regulatory stipulations, and most importantly affordable enough to attract users and for the science to get done^3^. To support this task, we asked what was the hourly fee for unsupervised (Supplementary Fig. 2, Question 21, “What is your average hourly fee for Unsupervised /Regular session?”), supervised (Supplementary Fig. 2, Q22, “What is your average hourly fee for Supervised /Assisted session?”) and training sessions (Supplementary Fig. 2, Question 24, “What is your average hourly fee for Training sessions?”). For example, The World View fee for Wide Field Microscopes for “unsupervised”, “supervised” and “training” was $10-30. In an earlier 2014 international microscopy survey ^9^, the instrument hourly rates trend was similar across the collective regions, however, within the regions themselves differences were also identified. In our current survey, we focused on the worldwide regions

We asked the question about average weekly usage and number of active users (Supplementary Fig. 2, Question 16, “What is the average weekly usage and total number of users per instrument in your facility?”) and percentage utilization (Supplementary Fig. 2, Question 18, “What percent of equipment is utilized by Internal academic users, External academic users, Commercial users, and Other”). This section is probably the most complex one to analyze as so many facilities have somewhat different business models, but it provides important comparative information on the user base composition and tool capacity/utilization per type of instrument that could help serve as a useful baseline.

We also queried respondents about how old instruments were handled (Supplementary Fig. 2, Question 33, “How are old instruments handled?”), service contracts (Supplementary Fig. 2, Question 34, “What is the source of funding for service contracts?”, Question 35, “Who services your Instruments?”, Question 36, “What percent of your high end equipment do you maintain on a service contract?”) and other service and technical related issues (Supplementary Fig. 2, Questions 35-42). Overall, this section should be very useful to core managers to understand how peer institutions handle this significant operating cost which also relates to up-time. Furthermore, it provides instrument manufacturer specific data and supplies a snapshot of how their instruments were performing. Interestingly, and strikingly, the greatest frequency of facilities that maintained high end equipment under contract (Question 36) at 76-100% with World View at 35% with Europe & Africa nearly 50% and Asia Pacific ~22%. It was also notable that the Asia Pacific peak frequency was <25% maintained under contract at 35% of facilities. This finding, in light of differences in funding sources (Question 13), is very interesting, but it also could reflect utilization levels, instrument complexity, purchase structures (extended warrantees), local equipment/service reliability and/or sales/service/support model between the regions, to name just a few.

#### Covid-19

Considering we are now well beyond the primary impacts of COVID-19 on core facilities, we still felt it worthwhile to memorialize and reflect on the collective experiences of our respondents (see Supplementary Fig. 2, Questions 44-53 and Supplementary Fig. 4 A-D). Some of the questions that we asked were what was the impact on utilization (e.g., Question 44 Supplementary Fig 4 B “During lockdown, how did COVID-19 impact the utilization of your facility?”) and personal protective equipment (PPE) requirements (Question 50, “What PPE are required to use your core?” Supplementary Fig. 4 D) the sterilization (e.g., Question 53 “Who sterilizes the equipment during COVID-19?”) and For example, the response to Question 44 (Supplementary Fig. 4 B) showed an impactful World View average of >40% decrease in utilization with The Americas and Europe & Africa having at least a 50% decrease while the Asia Pacific reported an ~20% decrease. Interestingly, and perhaps unsurprisingly, one of the biggest technological levers during COVID-19 (and beyond) was the use of some form of online training with nearly 60% of facilities (World View) planning to incorporate. Indeed, a knock-on effect, while not captured by our study, we anecdotally observed microscope related companies and core managers themselves seemed to be more accepting and rapidly adopting remote demonstrations to support purchase decisions. We feel this remote training/demonstration is now having a positive impact on the efficiency, environmental impact and sustainability of cores. Additionally, despite being in the rearview mirror, these COVID-19 responses are an invaluable resource and our learnings may be applied for yet unknown challenges, disasters or other contingencies in the future and, of course, what would be done differently.

#### Applications/Methods being used within Core Facilities

**Figure 10:**
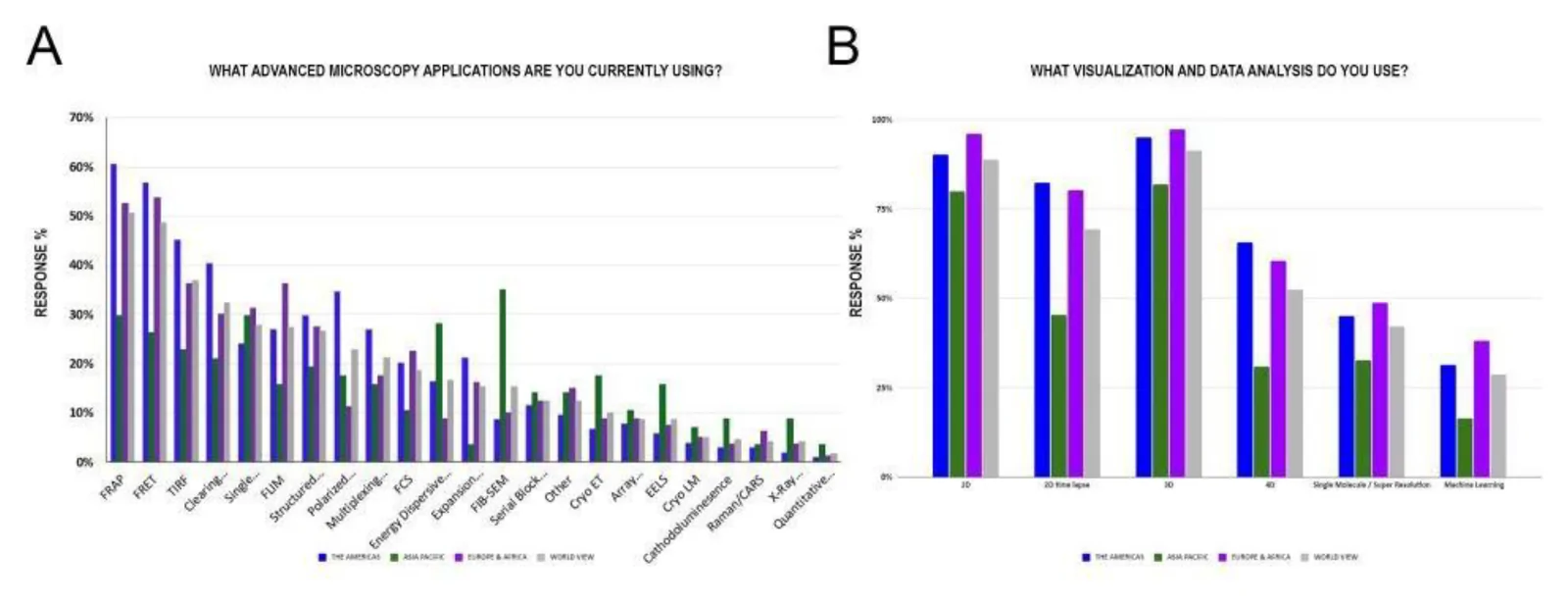
Advanced Microscopy Applications and Visualization/Data Analysis Approaches. (A) Advanced Microscopy Applications, (B) Visualization and Data Analysis.

To understand what type and relative levels of imaging approaches were being used, the survey looked at the different applications being leveraged in their facility (Fig. 10 A, Question 27, “What advanced Microscopy applications are you currently using?”). This showed that The America’s were generally doing more complex (e.g. FCS, FRAP, FRET, TIRFM) and newer applications (e.g. Clearing, Expansion, multiplexing microscopy) compared to the other regions. Europe & Africa also demonstrated a greater number of fluorescence techniques over the Asia Pacific which reported greater EM applications. The difference with Asia Pacific compared to the other regions could partly reflect that the respondent labs were more heavily involved with EM compared to Light Microscopy (LM) techniques and that the survey may not have reached as many LM labs, whereas the survey may not have reached as many EM labs in the non-Asia Pacific regions. A similar relationship was also observed for data analysis, with The Americas’ core facilities being more involved with the data analysis compared to Europe and Africa followed by Asia Pacific (Fig. 10 B, Question 28, “What Visualization and Data Analysis approaches do you use?”). Interestingly, at the time, World View use of Machine Learning was over 25% of respondents while Asia Pacific had ~15% of respondents. One explanation of the regional variation in data analysis, which can be labor-intensive, may reflect differences in what is typically offered as a service or technology access in the respective regions. Meaning, data analysis may be outsourced to different institutional resources or left to the user to tackle independently.

## Discussion and Conclusion

Our analysis focused on the regional commonalities and differences, which is complementary to the Schmidt survey^11^ that was focused more on the general international trends. As this survey shows there were many points of commonality and, of course, some differences between the regions. As our long-term goal is to develop a longitudinal study of microscopy cores, we wanted to benchmark where possible, a few prior published surveys with handful of questions and accordant answers that were substantially same.

As with the accompanying flow cytometry survey, we realized that several questions could have been better worded, for example, when asking for the number FTE in the core, as with the flow survey, we should have more clearly defined FTE and included the respondent. Phrasing will also be improved in the next survey to remove ambiguity in some questions and also give example. Thus, we would modify Question 6 “How many Staff members do you have? (excluding you)” to read “How many Staff members do you have (including you)” to make analysis much easier. Additionally, Question 7 “How many Full Time Equivalent (FTE) staff do you have? (excluding you eg. 3.25 FTE),” might be better worded as “How much total dedicated staff time supports your facility? (e.g., if you are an academic lead with 20% effort dedicated to the facility, this equals 0.2; if you have a part-time employee working 3 days a week, this equals 0.6, and if you work full-time/5 days a week, this equals 1.0, if you have all of these of these three, add them together).” In retrospect, these clarifications would minimize ambiguity on such important questions. One missed opportunity is asking whether the core facility included more than one type of platform, e.g. does your core also host flow cytometry, or another modality? It will also include new areas that develop, such as does your core have dedicated image analysis staff. We will also run a trial pilot survey across multiple different facilities from different geographical regions to identify any potential problems in advance and ease of analysis. It may be more time consuming upfront, but will save a significant effort later on.

There are many excellent core facility resources and surveys to be sure^5,8,9,12–16^, but relatively few that were focused specifically on Microscopy, fewer that cover multiple imaging forms (e.g. light, electron, x-ray, atomic force) and even fewer that were worldwide. For this comparison, we relied on an initial survey taken in 2011^9^ which encapsulated data from 175 respondents which effectively reflects a 10-year gap in survey completion. We also compared where possible a 2020 survey in the UK^1^ that included 46 light microscopy facilities and was chronologically close to our 2021 survey reported herein. While the 2011 prior survey lacked the same geographical breadth and distribution (63% North America, 29% Europe and 8% Asia Pacific), we still felt it worthwhile to draw some comparisons with key overlapping questions and other surveys were also included and compared. Interestingly, the Asia Pacific respondents in both surveys (Wallrabe^9^ and here) had a greater proportion of SEM and TEM systems (2011, 50% and 57.1%, 2021, 58% and 67%, respectively) compared to the World View (2011, 29.7% and 34.3%, 46% and 50%, respectively). Notably, all regions showed cores with an increase in the number of EM systems between these surveys. While not possible here, if the same labs could have been tracked in a longitudinal study, it would have been inciteful to know if this reflected a shift in core composition/focus or simply an increase in inclusion of EM lab respondents. Labs with both EM and Light Microscopy World View were 34.3% in 2011 and 28.9% in 2021 with only 1 EM ab of 26 did not report having some form of LM. Unsurprisingly, a much larger proportion of respondents had widefield and confocal microscopes in both surveys (e.g., confocal World View, 90% in 2011, 75% in 2021). Although curiously, there was a relative drop in confocal microscopes in our more recent survey which may correspond to the increase in EM cores as discussed above. To get a more granular perspective, we looked closely at the top advanced microscopy applications in these studies. FRAP, FRET, TIRF, Clearing Microscopy, Single Molecule, FLIM and Structured Illumination topped the list (rank ordered greatest to least) and ranged from 52-25% of respondents in 2021. In 2011, FRAP, FRET, Photo Activation, FLIM and Super Resolution (rank ordered greatest to least) and ranged from 67-45% of respondents. While there were some minor differences in advanced choices between the surveys (Clearing Microscopy added in 2021, Photo Activation removed in 2021 and Super Resolution in 2011 was split to Single Molecule and Structured Illumination) overall, there was remarkable similarity of the dominant techniques. Notably, EM tomography was the most popular EM Technique in 2011, while in 2021, Focused Ion Beam-SEM, Energy Dispersive Spectroscopy and Cryo Electron Tomography (rank ordered greatest to least) but ranged from 10-20%

Funding sources also had a very interesting trend, where almost all regions (except Canada) in 2011 derived about one-third of their operational funds from user fees, compared to 2021 where ~50% in The America’s, 45% in Europe & Africa and the Asia Pacific remained the same at about 33%. Did this reflect an increase in expectations/pressure for cost recovery over the last decade, or simply more organized or updated cores with increased usage/user-base that resulted in increased revenues? In comparison, for the UK light microscopy survey^1^, institutional support (43.3%) constituted a little larger portion of funding sources compared to user fees (39%). The user fees were not appreciably different than the 45% for Europe & Africa.

This survey serves as a baseline for the longitudinal studies to compare and contrast the global microscopy cores and to see what new technologies become mainstream, how funding mechanisms and costs change and how staff levels change over the coming years, to name a few. It was good to see Schmidt^11^ have very similar findings in a completely independent survey. Any future surveys hopefully will be combined and also informed from results here as well as adapting to changes in technology and core business models. For example, we found that there were questions that should have been asked and that will help inform us of the way future surveys will be worded. We certainly found that some questions may have been ambiguous, which became apparent when the data was analyzed. For example, Question 6, “How many Staff members do you have (excluding you)”? we believe some respondents did not take into account if they had one or more part-time employees, which could inflate our calculations and correlations of the number of staff relative to other metrics. Our intent for Question 7, “How many Full Time Equivalent (FTE) staff do you have (excluding you e.g. 3.25 FTE)?” was to accommodate part-time staff, but looking at inconsistencies in responses for both questions, suggested that the term “FTE” may not be familiar or commonly used in some geographic regions. Additionally, Question 10, “What is the annual operating cost of your Microscopy Facility?” and Question 11, “Does the total operating cost from the above question include staff salary?” while provided useful reporting ranges, left sufficient vagueness when correlating with overall facility usage and equipment type and number. Specifically, we believe many core facilities, particularly smaller ones, have budgeting models that often exclude some or all salary, overhead, depreciation and/or core managers do not have detailed budget planning that include all costs that many larger cores have.

What was striking overall was how similar most responses were between the regions. Where notable differences were observed, this could in part reflect the respondents within those regions, especially within the Asia Pacific region that resulted in proportionally more materials and electron microscopy core facilities. But even in such cases, the results were remarkably similar in most aspects surveyed. Of course, we found that there were questions that should have been asked and this will help inform us how to improve wording in the next survey. Furthermore, since we concomitantly surveyed, with substantially identical questions, our closely aligned flow cytometry facility colleagues (and often incorporated as part of microscopy cores) we refer you to the companion article within this issue^10^ for direct comparisons of these 2 surveys. Finally, since the survey was so data rich, it was not possible to compare and contrast every possibility within the scope of this manuscript. For this reason, we have made all the survey data responses anonymized (only country identifier provided) and available as a spreadsheet accessible to the public (Supplementary Table 1). This will allow for the microscopy community opportunities for further analysis and data to be mined for specific questions that may be useful for other purposes.

Finally, for those interested in suggestions for a trimmed version of questions for your own future survey that still would allow many important comparisons with those included herein, we color-coded the question list (Supplementary Fig. 2 – slides 2-4) with “Black” being high priority, “Orange” being medium priority and “Red” being low priority. Driving our lower prioritization rating were a series of Covid questions, questions that had very high uniform and/or obvious responses (e.g., Question 23, “Do you offer training for users?”), or questions that we considered important but required much more time effort for the respondents (e.g., Question 21, “What is your average hourly fee for Unsupervised/Regular session?”).

## Supplementary Material

Supplementary Fig 1

Supplementary Fig 2

Supplementary Fig 3

Supplementary Fig 4

Supplementary Table 1

Supplementary Figures Legends
