# Supplementary material for: Global Survey of Microscopy Core Facilities
Source: J Microsc. Author manuscript; Available in PMC 2026 Oct 16. (PMC7619576; doi:10.1111/jmi.70097)
Supplement: Supplementary file 1 [file EMS214924-supplement-Supplementary_Figures_Legends.docx]

631

632

633	**Supplementary** **Figure** **1**. Microscopy Survey Demographic Country Heatmap. This global

634	Microscopy core facility survey included 241 participants from 24 countries.

635	See Figure 1 for the country allocation into the 3 distinct geographical regions, “The Americas”,

636	“Europe & Africa” and “Asia Pacific”.

637

638

639

640

641

642

643

644

645

**Supplementary** **Figure** **2**. See Supplementary PowerPoint with All Questions as Charts

646

647	**Supplementary** **Figure** **3**	. Staff vs How many instruments (A) The Americas, (B) Asia Pacific,

648	(C) Europe & Africa, (D) World View

649

650

651

652

653

654

655

656

657

658

659

660	**Supplementary** **Figure** **4**	**.** Impact of COVID. (A) Utilization in prior 3 years, (B) Utilization

661	during lockdown, (C)Training during COVID, (D) PPE used during COVID.
